# Molecular basis for pericyte-induced capillary tube network assembly and maturation

**DOI:** 10.3389/fcell.2022.943533

**Published:** 2022-08-22

**Authors:** Scott S. Kemp, Prisca K. Lin, Zheying Sun, Maria A. Castaño, Ksenia Yrigoin, Marlena R. Penn, George E. Davis

**Affiliations:** Department of Molecular Pharmacology and Physiology, Morsani College of Medicine, University of South Florida School of Medicine, Tampa, FL, United States

**Keywords:** pericytes, endothelial cells, extracellular matrix, capillary assembly, basement membrane deposition

## Abstract

Here we address the functional importance and role of pericytes in capillary tube network assembly, an essential process that is required for vascularized tissue development, maintenance, and health. Healthy capillaries may be directly capable of suppressing human disease. Considerable advances have occurred in our understanding of the molecular and signaling requirements controlling EC lumen and tube formation in 3D extracellular matrices. A combination of SCF, IL-3, SDF-1α, FGF-2 and insulin (“Factors”) in conjunction with integrin- and MT1-MMP-induced signaling are required for EC sprouting behavior and tube formation under serum-free defined conditions. Pericyte recruitment to the abluminal EC tube surface results in elongated and narrow tube diameters and deposition of the vascular basement membrane. In contrast, EC tubes in the absence of pericytes continue to widen and shorten over time and fail to deposit basement membranes. Pericyte invasion, recruitment and proliferation in 3D matrices requires the presence of ECs. A detailed analysis identified that EC-derived PDGF-BB, PDGF-DD, ET-1, HB-EGF, and TGFβ1 are necessary for pericyte recruitment, proliferation, and basement membrane deposition. Blockade of these individual factors causes significant pericyte inhibition, but combined blockade profoundly interferes with these events, resulting in markedly widened EC tubes without basement membranes, like when pericytes are absent.

## Introduction

A fundamental requirement for the development and maintenance of the vascular system is the interaction between endothelial cells (ECs) and mural cells, which can be pericytes or vascular smooth muscle cells (VSMCs) (Adams and Alitalo, 2007; Armulik et al., 2011; Stratman and Davis, 2012; Bowers et al., 2016; Payne et al., 2020). The vasculature, which is exposed to blood, has an EC lining and on its abluminal side, pericytes or vascular smooth muscle cells are present. In addition, the vascular basement membrane, which has unique components and features compared to epithelial, skeletal muscle or cardiac muscle basement membranes, is found underneath the EC surface and is typically in between the ECs and mural cells (Yurchenco et al., 2004; Davis and Senger, 2005; Stratman et al., 2009a; Senger and Davis, 2011; Yurchenco, 2011; Stratman and Davis, 2012; Davis et al., 2015; Pozzi et al., 2017). Basement membranes can be continuous or discontinuous depending on the tissue location of the vasculature (Eble and Niland, 2009; Augustin and Koh, 2017). Thus, there is an intrinsic polarity to the organization of the vascular wall with the EC layer, an underlying basement membrane, pericytes underlying the basement membrane in capillaries, and then a switch to vascular smooth muscle cells (organized in concentric layers) as arteries and veins increase in size. From a cellular and molecular perspective, how do these vascular assemblies occur and what growth factors, peptides, extracellular matrices (ECM), and signaling events are required for the different steps controlling these processes? Considerable progress has been made to elucidate these key steps, however, many unresolved questions remain. In this review, we will direct our focus to the functional role of pericytes in their ability to control the establishment and maintenance of capillary networks and, also the growth factor and molecular requirements necessary for the assembly of branched arrays of EC-lined tubes, a necessary cellular structural network that interacts with mural cells to generate the different types of blood vessels.

### Key principles governing capillary network assembly

**FIGURE 1:**
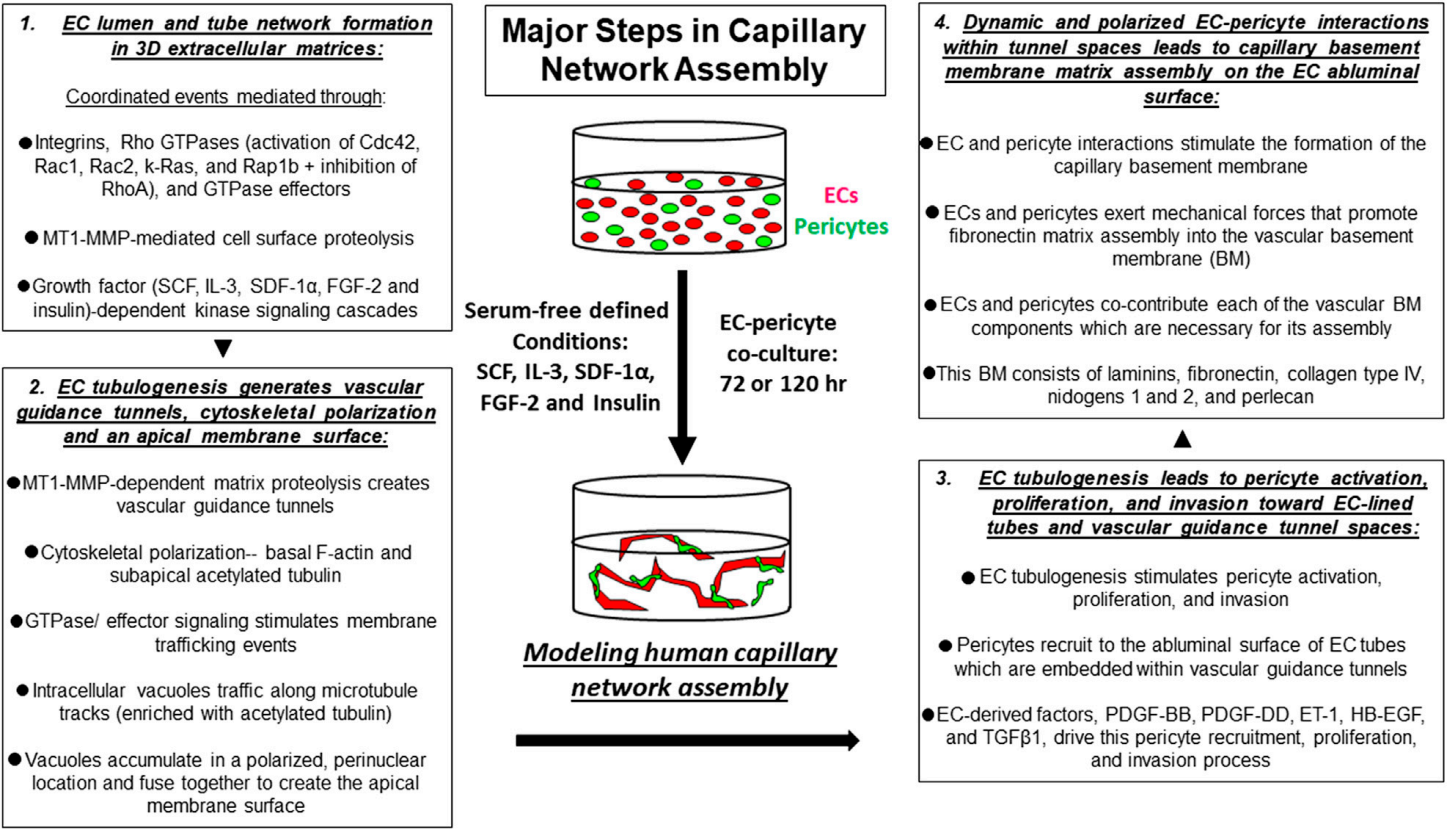
Key steps controlling capillary tube network assembly in 3D extracellular matrices. *In vitro* model systems have been developed to mimic the normal process of human capillary network assembly in a 3D matrix environment. Using this defined system, serum-free conditions, and the addition of a combination of five growth factors (“Factors”) consisting of SCF, IL-3, SDF-1α, FGF-2 and insulin allows for human ECs and pericytes to co-assemble into capillary networks over time.

The formation of capillaries requires two cell types: ECs and pericytes. Their interactions are necessary for the capillary assembly process. Capillaries play major roles in tissue development, maturation and maintenance, and deliver signals beyond oxygen and nutrients to tissues to control organismal health. Considerable progress has been made in our understanding of capillary biology. This has occurred in part due to the development of sophisticated *in vitro* models of human capillary assembly in three-dimensional (3D) matrices (Nakatsu et al., 2003; Koh et al., 2008b; Nakatsu and Hughes, 2008; Stratman et al., 2009a; Stratman et al., 2011). Two key 3D interstitial matrices (i.e. collagen type I and fibrin/fibronectin) are the best matrices (Montesano et al., 1983; Montesano et al., 1987; Davis and Camarillo, 1996; Bayless et al., 2000; Bayless and Davis, 2003; Aplin et al., 2008; Koh et al., 2008b; Nakatsu and Hughes, 2008), to strongly support and drive vascular morphogenesis and, also EC-pericyte tube co-assembly leading to the formation of capillary tube networks. An important fibrin model developed by the laboratory of Christopher Hughes has also been utilized (Nakatsu et al., 2003; Nakatsu and Hughes, 2008). Using a modified design of this system, flow forces can be applied to the EC tube networks in a 3D matrix environment and compared to lack of flow (Wang et al., 2016; Wang et al., 2017). Another significant advance has been the establishment of human EC tube formation, EC sprouting, and EC-pericyte tube co-assembly bioassay systems in both 3D collagen and fibrin matrices under serum-free defined conditions (Stratman et al., 2009a; Stratman et al., 2011; Smith et al., 2013; Bowers et al., 2015; Bowers et al., 2016; Bowers et al., 2020) (Figure 1). These latter models, developed by our laboratory, are allowing us to define the growth factor requirements and signaling events that are necessary for EC tubulogenesis, EC sprouting behavior, pericyte recruitment to EC tubes and pericyte-induced EC tube maturation, which includes vascular basement membrane matrix deposition (Bowers et al., 2016) (Figure 1). A critical point is that the elucidation of the underlying biology of capillary network assembly is essential to understand the pathogenesis of key human disease states, where capillary disassembly or dysfunction is a central issue. Such diseases include diabetes, malignant cancers, vascular malformations, ischemic injuries such as myocardial infarction, stroke, and the progression to chronic kidney disease, hypertension, heart failure, sepsis, and neurodegeneration, including Alzheimer’s disease (Morikawa et al., 2002; Muris et al., 2013; Karaca et al., 2014; Wetzel-Strong et al., 2017; Afsar et al., 2018; Erdener and Dalkara, 2019; Zeng and Chen, 2019; Kida, 2020; Horton and Barrett, 2021; Picoli et al., 2021). In addition, recent studies have strongly implicated capillary loss as an important factor contributing to aging which may accelerate the process (Chen et al., 2021a; Chen et al., 2021b; Grunewald et al., 2021).

**FIGURE 2:**
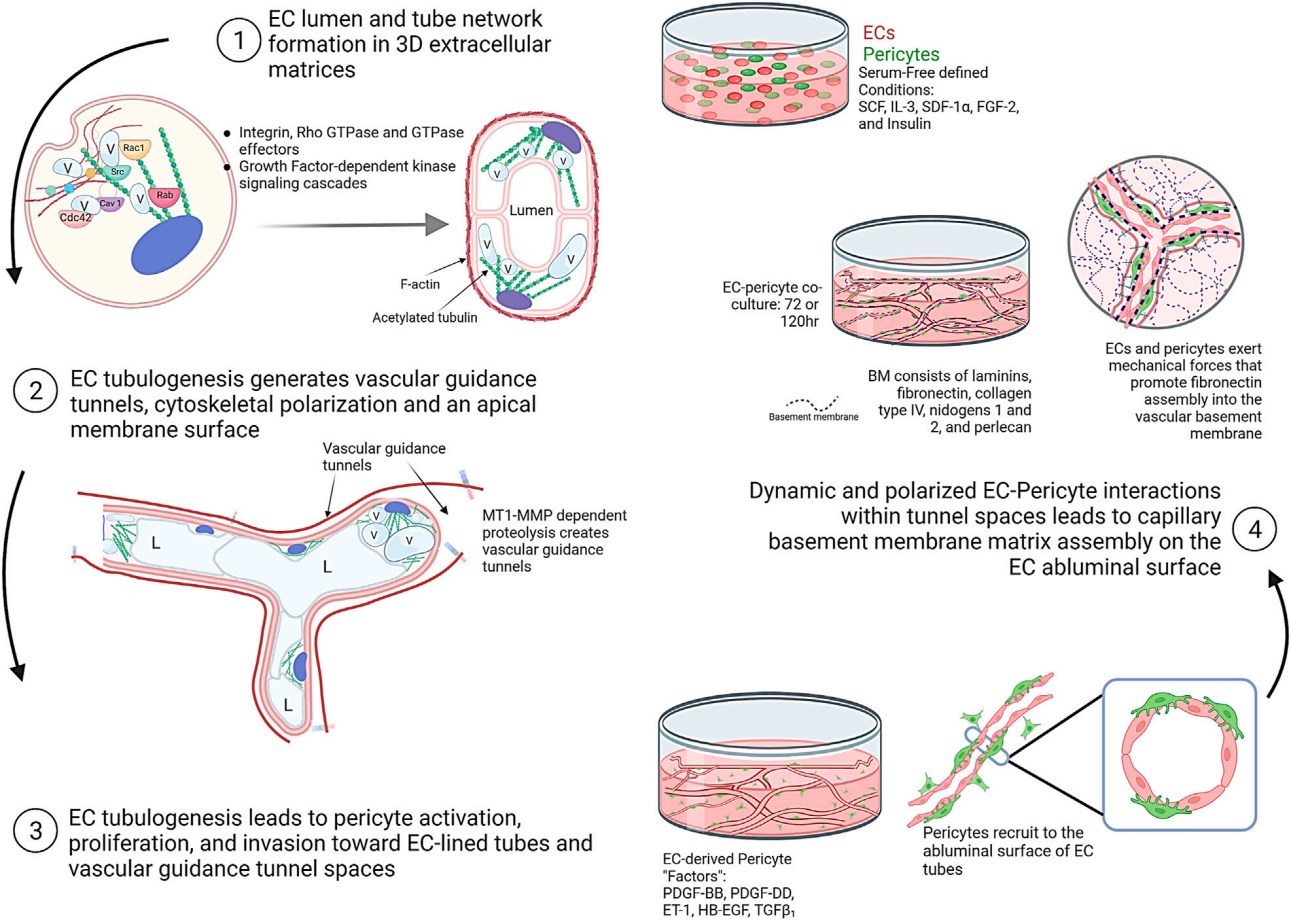
Schematic illustrations showing the fundamental and interconnected steps controlling the assembly of human capillary tube networks. Four key steps are depicted showing the ability of ECs to form lumens, tube networks, and vascular guidance tunnels following by pericyte activation, invasion, and recruitment to these EC-lined tubes in a polarized, abluminal position. This latter step requires the indicated EC-derived factors which consist of PDGF-BB, PDGF-DD, ET-1, TGFβ1, and HB-EGF. EC-pericyte dynamic and polarized interactions within tunnel spaces leads to capillary basement membrane deposition between the two cell types. This figure was prepared on the website, Biorender.com.

The key steps in capillary tube network assembly occur as follows. During developmental vasculogenesis, where ECs assemble *de novo* from individual and small clusters of cells, EC interactions with extracellular matrix *via* integrins leads to cell elongation, intracellular vacuolation and fusion, lumen formation, and multicellular tube assembly over time (Davis et al., 2011) (Figures 1, 2). EC lumen formation is best visualized using histologic cross-sections or by confocal microscopic analysis (Davis and Camarillo, 1996; Koh et al., 2008b; Davis et al., 2011; Stratman et al., 2011; Smith et al., 2013). An important point is that ECs on their own are fully capable of forming lumens and multicellular tube networks in a 3D matrix environment. Suggestions that other cell types such as mural cells are required in conjunction with ECs for this process are not supported by extensive *in vitro* data. ECs sprout and form lumens and tubes in response to specific growth factor combinations (*see* later on), and then attract mural cells such as pericytes to create capillary networks. In our model systems under defined serum-free conditions, pericytes on their own show minimal migratory or proliferative behavior in 3D matrices (Stratman et al., 2010; Kemp et al., 2020). However, in the presence of ECs, the pericytes become activated, elongate, migrate, and invade in 3D matrices to reach the EC tube abluminal surface (Stratman et al., 2010; Kemp et al., 2020). They then migrate along this surface in a polarized fashion to form the capillary basement membrane in conjunction with ECs (which deposits abluminally between the two cell types) (Stratman et al., 2009a; Stratman and Davis, 2012; Bowers et al., 2020; Kemp et al., 2020). This pericyte response to the presence of ECs is due to EC production of key pericyte factors that are necessary for pericyte recruitment, proliferation, and pericyte-induced basement membrane deposition (*see* later on) (Stratman et al., 2010; Kemp et al., 2020).

### Molecules and signaling pathways controlling EC lumen and tube formation

In order to form functional capillary networks, EC lumen and tube formation is necessary to allow for connection to the systemic circulation and perfusion of these blood vessels. Further maturation and stabilization of these blood vascular tubes occurs due to this perfusion (Iruela-Arispe and Davis, 2009). For example, EC Notch activation is stimulated downstream of flow forces that induce EC differentiation into the arterial lineage (Lawson et al., 2001), a process which is also accompanied by reduced EC proliferation (Fang et al., 2017). Interestingly, past DNA microarray studies examining differential gene expression during EC tube formation revealed marked downregulation of cell cycle genes associated with proliferation (Bell et al., 2001). Furthermore, recent studies have confirmed this finding by showing that EC tubulogenesis stimulated *in vitro* using defined growth factors leads to cessation of EC proliferation (Bowers et al., 2020). Interestingly, although EC proliferation is suppressed during tubulogenesis, addition of pericytes leads to pericyte proliferation, a response that depends on ECs and their secretion of key growth factors and peptides, including PDGF-BB, PDGF-DD, HB-EGF, and endothelin-1 (ET-1) during capillary assembly (Stratman et al., 2010; Bowers et al., 2020; Kemp et al., 2020).

### Critical importance of MT1-MMP in EC lumen formation, tubulogenesis and vascular guidance tunnel formation

Considerable effort has occurred, and great progress has been made toward elucidating the molecular basis for EC lumen and tube formation. EC tube formation is a 3D matrix-specific process that depends on integrin-extracellular matrix (ECM) interactions as well as signaling coordination with specific growth factor-dependent pathways and concurrent MT1-MMP-dependent ECM proteolysis (Stratman et al., 2009b; Sacharidou et al., 2010; Davis et al., 2011; Davis et al., 2015; Bowers et al., 2016). An important finding was the demonstration that EC tubulogenesis requires the creation of vascular guidance tunnels, which are matrix-free spaces that are generated by MT1-MMP-dependent proteolysis of 3D matrices (Stratman et al., 2009b; Sacharidou et al., 2010). Blockade of MT1-MMP, using various chemical or protein MMP inhibitors, completely interferes with vascular guidance tunnel formation. In addition, blockade of EC lumen formation using inhibition of key EC lumen formation regulators, such as protein kinase C epsilon or Src kinases, also completely interferes with tunnel formation (Stratman et al., 2009b). Thus, vascular guidance tunnels are generated during EC tubulogenesis, and interference with their formation completely inhibits the lumen and tube formation process (Stratman et al., 2009b). Once vascular guidance tunnels have been formed, ECs and EC tube networks can migrate and remodel in an MT1-MMP-independent manner in 3D matrices (Stratman et al., 2009b). Another key point is that vascular guidance tunnels are required for the next steps in blood vessel maturation that involve the recruitment of pericytes to the EC tube abluminal surface within the tunnel spaces (Stratman et al., 2009a). The vascular guidance tunnel spaces allow for EC tube remodeling and pericyte motility along the abluminal surface, and these cells together assemble the capillary basement membrane matrix in a polarized manner between the two cell types (Stratman et al., 2009a; Stratman and Davis, 2012) (Figures 1, 2).

### Integrin-dependence for EC tubulogenesis and ECM-dependent EC tube maturation events

Integrin-ECM interactions in 3D matrices are required for the EC tubulogenic process and key integrins that have been shown to stimulate this process are α2β1 and α5β1, which are collagen and fibronectin receptors, respectively (Davis and Camarillo, 1996; Bayless et al., 2000; Bayless and Davis, 2003; Smith et al., 2013). Fibronectin- and collagen type I-rich interstitial matrices appear to be the major ECM stimuli that drives vascular morphogenesis during vascular development and postnatal life (Senger, 1996; Davis and Senger, 2005; Astrof et al., 2007; Hynes, 2007; Astrof and Hynes, 2009; Senger and Davis, 2011). Both α5β1 and α2β1 have been strongly implicated *in vivo* as well as *in vitro* as the major integrin stimulators of EC tubulogenesis (Davis and Camarillo, 1996; Bayless et al., 2000; Francis et al., 2002; Senger et al., 2002; Zweers et al., 2007; San Antonio et al., 2009). The αvβ3 integrin, an RGD-binding integrin, has been implicated during tissue injury responses including that occurring during tumor angiogenesis (Brooks et al., 1994). This is due to the fact that it can interact with plasma-derived integrin ligands such as vitronectin and fibronectin (Bayless et al., 2000), injury-induced molecules such as osteopontin (Bayless et al., 1997), or by interacting with denatured collagens (generated during tissue damage) through the exposure of matricryptic RGD sites (Davis, 1992; Davis et al., 2000). Integrins are also important during EC tube maturation responses where ECs interact with basement membrane components (Stratman et al., 2009a). Key EC integrins with affinity for basement membrane matrix proteins include α1β1, α2β1, α3β1, α5β1, α6β1, and αvβ3 (Senger and Davis, 2011; Yurchenco, 2011; Hohenester and Yurchenco, 2013; Davis and Kemp, 2022). Pericytes also express basement membrane binding integrins including α1β1, α2β1, α3β1, α4β1, α5β1, α6β1, and αvβ3 (Stratman et al., 2009a). In contrast to the role of interstitial matrices in stimulating vascular morphogenesis, it appears that basement membrane matrices, have a distinct role which is to affect vascular maturation and stabilization (Stratman et al., 2009a). For example, combined blockade of α3β1 and α6β1, two laminin receptors, inhibited vessel maturation with widened tubes, but did not inhibit the tube formation process itself (Stratman et al., 2009a). More work is needed to investigate the specific functions of these different EC and pericyte integrins during EC tube maturation events, through their interactions with basement membrane matrix components.

**FIGURE 3:**
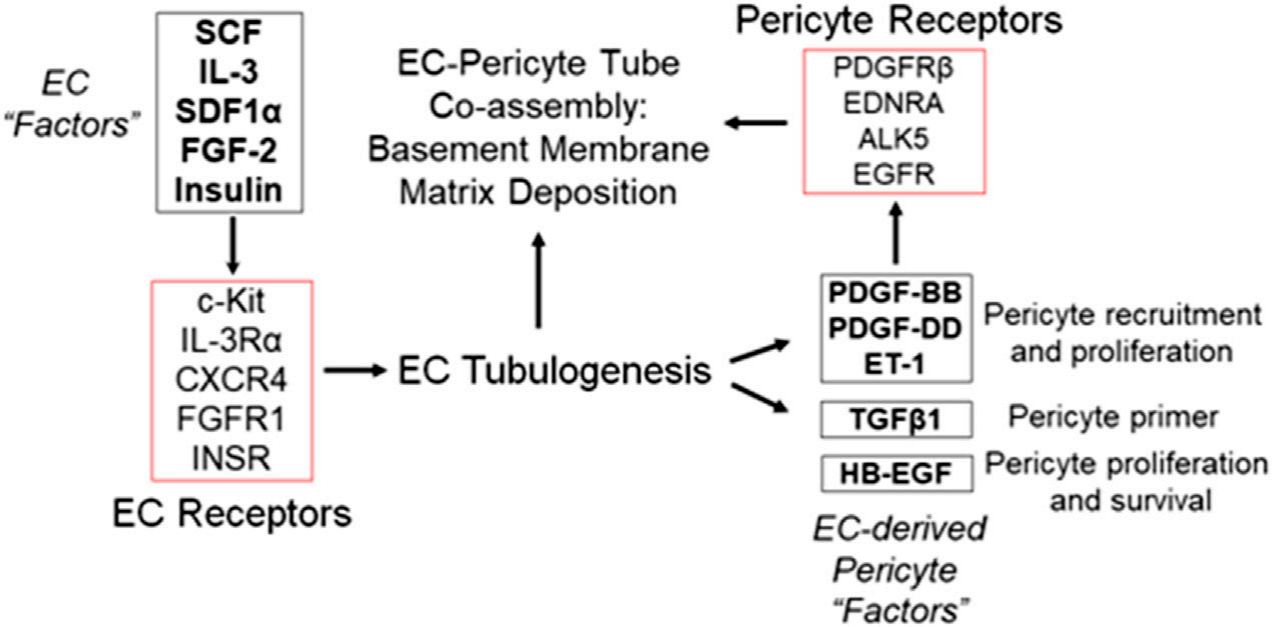
Defining the growth factors and peptides that are required for both ECs and pericytes to co-assemble into human capillary tube networks. **(A)** Using a defined serum-free system in 3D collagen or fibrin matrices, the growth factor and peptide requirements for human EC-pericyte tube co-assembly were determined. A five growth factor combination consisting of SCF, IL-3, SDF-1α, FGF-2 and insulin (“Factors”) has been shown to stimulate all aspects of vascular morphogenesis including EC lumen and tube assembly as well as sprouting behavior. Additional work demonstrated a requirement for ECs in stimulating pericytes to recruit to tubes, proliferate, and induce basement membrane deposition. Five EC-derived factors including PDGF-BB, PDGF-DD, ET-1, TGFβ1, and HB-EGF were found to work together to promote these events and their functional roles during this process are indicated.

Integrin-ECM interactions in conjunction with growth factor receptor activation leads to activation of signal transduction that is necessary for the ability of ECs to form lumens, sprout, and form branched networks of tubes (Somanath et al., 2009; Davis et al., 2011; Bowers et al., 2016). ECs do not proliferate during this morphogenic response and yet signal through the Ras/Map kinase pathway (typically, a mitogenic signaling pathway) to selectively stimulate EC lumen and tube formation and not proliferation. Using defined serum-free conditions, our laboratory discovered a five growth factor combination (i.e. “Factors”) that is required for human ECs to undergo tube morphogenesis and sprouting behavior in either 3D collagen or fibrin matrices (Stratman et al., 2011; Smith et al., 2013; Bowers et al., 2015; Bowers et al., 2016). These growth factors are stem cell factor (SCF), interleukin-3 (IL-3), stromal-derived factor (SDF)-1α, fibroblast growth factor (FGF)-2, and insulin (Stratman et al., 2011; Bowers et al., 2016) (Figures 1–3). We have yet to identify any other combination of growth factors or peptides that stimulate these processes (Bowers et al., 2016). Addition of VEGF alone, or VEGF in combination with FGF-2 fails to stimulate EC tube formation under these defined conditions (Stratman et al., 2011). However, VEGF can act as an upstream priming stimulus, which enhances EC responsiveness to the addition of the downstream “Factors” and, thus, stimulates EC tubulogenesis to a greater extent than the influence of the “Factors” alone (Stratman et al., 2011; Bowers et al., 2020) (*see* later on). In part, this is due to the ability of VEGF to induce EC expression of c-Kit, IL-3Rα, and CXCR4, which are receptors for three of the “Factors,” SCF, IL-3, and SDF-1α, respectively (Bowers et al., 2020).

### Role of small GTPases and their effectors, and key kinase cascades in EC lumen and tube formation

Coordinated integrin- and “Factor”- mediated signaling in ECs in conjunction with cell surface MT1-MMP-induced ECM proteolysis leads to EC lumen and tube formation as well as sprouting behavior (Davis et al., 2011; Sacharidou et al., 2012; Davis et al., 2015; Bowers et al., 2016). In the former case, multiple small GTPases of the Rho GTPase family have been shown to be critical and activated during EC lumen formation: these include Cdc42, Rac1, Rac2, k-Ras, and Rap1b (Bayless and Davis, 2002; Koh et al., 2008a; Hoang et al., 2011b; a;Barry et al., 2015; Norden et al., 2016). Key effectors (and several binding partners of these effectors) of these GTPases including Pak2, Pak4, IQGAP1, MRCKβ, Rasip1, β-Pix, GIT1, RhoJ, CCM1, and CCM2, are also necessary for the lumen formation process (Koh et al., 2008a; Kleaveland et al., 2009; Koh et al., 2009; Whitehead et al., 2009; Lampugnani et al., 2010; Xu et al., 2011; Yuan et al., 2011; Sacharidou et al., 2012). Other important regulatory or effector kinases that control the EC lumen formation signaling process are protein kinase C epsilon (PKCε), Src family kinases (Src, Fyn, Yes), Raf kinases, Mek kinases and Erk kinase (Alavi et al., 2003; Koh et al., 2009; Sacharidou et al., 2012; Lanahan et al., 2013; Kim et al., 2017). In very recent work, several studies have also implicated Rab GTPases (i.e. Rab27a, Rab8a, Rab11a, Rab3a, Rab3b, Rab35) and Ral (i.e. RalA, RalB) GTPases, which control vesicle trafficking and exocytosis in the EC lumen formation process (Norden et al., 2020; Francis and Kushner, 2021). In total, these molecules and signaling events control the major steps in EC lumen and tube formation. These steps are: *1*) to stimulate membrane trafficking events to create and add membrane to the developing apical surface *via* intracellular vacuole formation, vacuole trafficking and coalescence (Davis and Camarillo, 1996; Bayless and Davis, 2002; Kamei et al., 2006; Kim et al., 2017; Norden et al., 2020); *2*) to generate vascular guidance tunnels that are mediated by localized MT1-MMP-dependent matrix proteolysis (Stratman et al., 2009b); and *3*) to create cytoskeletal polarization with F-actin distributed basally and post-translationally modified tubulins such as acetylated tubulin distributed in a subapical region which facilitates the trafficking of intracellular vacuoles to the apical membrane surface and then facilitates apical membrane stabilization (Kim et al., 2013; Kim et al., 2017) (Figures 1, 2). Thus, the purpose of the EC tubulogenic signaling cascade is to stimulate the formation of EC-lined tube networks. The establishment of these tubes, then, is coordinated with the recruitment of mural cells, such as pericytes, which are required for capillary maturation and stabilization (Stratman et al., 2009a; Bowers et al., 2020; Kemp et al., 2020).

### Defining the growth factors and peptides that are necessary for pericyte recruitment, proliferation and pericyte-induced EC basement membrane deposition during capillary network assembly

A central question and issue in the assembly of capillary tube networks is what growth factors, peptides or other molecules control the recruitment of pericytes to the assembling EC tubes. There are different degrees of pericyte recruitment within capillaries depending on the tissue type, which is most accentuated in the brain, where higher percentages of pericytes (relative to ECs) are observed compared to peripheral tissues (Armulik et al., 2011). A series of genetic knockout studies in mice and zebrafish suggest an important role for PDGF-BB, and its receptor, PDGFRβ, which is highly expressed by pericytes (Lindahl et al., 1998; Hellstrom et al., 2001; Nystrom et al., 2006; Stratman et al., 2017) in this process. Other studies using knockout technology have also implicated TGFβ and HB-EGF in EC-mural cell interactions during the development of the vasculature (Oshima et al., 1996; Iivanainen et al., 2003; Carvalho et al., 2004; Lebrin et al., 2005; Ramsauer and D'Amore, 2007; ten Dijke and Arthur, 2007). The use of a defined 3D *in vitro* model to address these questions is critical because of the complexity of a whole animal. For example, distinguishing biological effects such as mural cell proliferation, survival, or recruitment to EC tubes can be difficult and subject to interpretation *in vivo*, while these issues can be more readily resolved *in vitro*.

To address these issues, studies were performed using a serum-free defined EC morphogenesis model using the “Factors,” whereby EC tubulogenesis occurs and pericytes recruit to these tube networks over a 3–5 day period in either 3D collagen or fibrin matrices (Stratman et al., 2009a; Smith et al., 2013; Bowers et al., 2020; Kemp et al., 2020). Pericytes, which were labeled with GFP, to distinguish them from ECs, were seen to become elongated and were also observed to divide during this EC-pericyte tube co-assembly process. In the initial set-up of this assay system, pericytes were seeded at a density of 20% of pericytes relative to ECs, which was optimal. Over time, they were seen to reach the EC tube surface, where 80% or greater of the pericytes were observed to recruit and elongate on the EC tubes by 72 h and beyond. Using these models, immunostaining of gels with antibodies directed to collagen type I or fibrinogen to visualize the ECM, led to the demonstration of vascular guidance tunnel networks. Both the EC-lined tubes and the recruited pericytes were located inside the tunnel spaces (Stratman et al., 2009a; Smith et al., 2013). Real-time movies revealed the dynamic nature of the EC-pericyte tube co-assembly process, and once pericytes had recruited to the EC tubes, they could be observed to be migrating up and down the tube networks, suggesting that they are migrating along the abluminal tube surfaces within the tunnel spaces. Interestingly, occasional pericytes were observed migrating in between tubes and leaving one tube and ending up along another tube surface. Additionally, the presence of pericytes markedly alters the outcome of the EC tube morphogenic process. As EC only tubes progressively get wider and shorter in length over time, pericyte recruitment to tubes leads to maintenance of the initial elongated tube structure with narrow diameters (Stratman et al., 2009a). A key difference between these two conditions, is that EC-pericyte co-cultures markedly deposit vascular basement membrane matrices on the EC abluminal surface, while EC only cultures do not (Stratman et al., 2009a). This conclusion was confirmed using transmission electron microscopy and immunofluorescence microscopy. In the latter case, immunostaining was performed using non-permeabilized (i.e. in the absence of detergent) cultures so that only extracellular deposition of basement membrane components was examined (Stratman et al., 2009a; Smith et al., 2013).

Nuclear GFP-labeled pericytes were utilized to address the role of specific growth factors or peptides affecting pericyte behavior during capillary assembly. These labeled pericytes were seeded in the presence of the EC “Factors” without or with added ECs. Pericytes did not migrate in 3D collagen matrices in the absence of ECs as evaluated by real-time movies imaged under fluorescence where nuclear tracking was assessed over time as a measure of cell motility (Stratman et al., 2010; Kemp et al., 2020). In contrast, when ECs were added with the pericytes, marked motility was observed over time, as they recruit to the EC tube surfaces (Stratman et al., 2010; Kemp et al., 2020). This pericyte motility is actually an invasion response, as an MT-MMP is required for invasion and motility of these cells in 3D matrices (i.e. addition of the MT-MMP inhibitor, GM6001, completely blocks invasion) (Kemp et al., 2020). Thus, EC-derived factors are critical for the pericyte response to EC tubes during capillary formation.

### Identification of EC-derived factors controlling pericyte recruitment, proliferation and pericyte-induced EC tube maturation events

To assess the direct influence of individual EC-derived factors, the ability of 23 different EC-derived factors to affect pericyte invasion of 3D collagen matrices (in the absence of ECs) was examined. Of these, three of them had a strong ability to drive pericyte invasion and they were PDGF-BB, PDGF-DD, and ET-1 (Kemp et al., 2020). When these three factors were combined, even greater stimulation of motility and invasion occurred, and mimicked pericyte invasion of 3D matrices when ECs are present (Kemp et al., 2020). In addition, these factors could stimulate pericyte proliferation along with HB-EGF (which did not directly stimulate pericyte invasion). Interestingly, TGFβ treatment was found to act as an upstream priming stimulus which enhances the ability of PDGFs and ET-1 to stimulate pericyte invasion (Kemp et al., 2020).

An important question was to examine the individual and collective influence of these five EC-derived factors on pericyte interactions with ECs, and the capillary maturation process. Individual blockade of each molecule or its receptor had significant effects on pericyte recruitment in the co-cultures. For example, neutralization of individual factors such as PDGF-BB with blocking antibodies only partially affected pericyte recruitment and proliferation (Kemp et al., 2020). In contrast, neutralization of all five factors in combination resulted in dramatic blockade of pericyte recruitment, such that essentially no recruitment or proliferation of pericytes occurred (Kemp et al., 2020). Also, EC tube formation was not blocked by the addition of these “Pericyte Factor” neutralizing antibodies. Under these conditions, EC tube formation behaved as if pericytes were never added. The EC tubes became markedly widened and shortened in length over time, just like what occurs when ECs are cultured to form tubes by themselves (Kemp et al., 2020). Two different sets of inhibitory cocktails were employed in these studies. One set was pharmacologic agents that were directed at the pericyte receptors, PDGFRβ, endothelin receptor A (EDNRA), EGFR and Alk-5, a TGFβ receptor (i.e. CIGS drug combination). When these agents were added by themselves, a partial inhibitory effect on pericyte recruitment and proliferation were observed, but when combined, there was a marked inhibitory effect (Kemp et al., 2020). The second set of agents utilized neutralizing antibody or receptor trap reagents blocking each of the growth factors. The same conclusions were observed where individual blocking antibodies showed partial effects, but when they were added in combination (along with the EDNRA inhibitor), profound inhibitory effects were observed with no pericyte recruitment or proliferation (Kemp et al., 2020). In addition, both sets of these combined inhibitory agents completely blocked pericyte-induced basement membrane matrix assembly around the EC tube networks (Kemp et al., 2020). This novel study demonstrated that at least five different EC-derived factors (PDGF-BB, PDGF-DD, ET-1, HB-EGF, and TGFβ1) are secreted by ECs during tubulogenesis to drive the pericyte recruitment process, and thus, control capillary tube network assembly and maturation (Kemp et al., 2020) (Figure 3).

### VEGF priming leads to accelerated “Factor”-dependent EC tubulogenesis, pericyte recruitment, proliferation, and basement membrane matrix deposition during capillary network assembly

There is considerable data in the literature demonstrating a functional role for VEGF and its receptor, VEGFR2, in tissue vascularization responses *in vivo* in multiple species (Ferrara et al., 1996; Lawson et al., 2002; Lanahan et al., 2013). Of great interest are the findings discussed above showing that VEGF does not directly control EC tube formation or sprouting behavior as a direct acting factor, even when added in combination with FGF-2, using serum-free defined systems in either 3D collagen or fibrin matrices (Stratman et al., 2011; Bowers et al., 2020). In contrast, when VEGF is added to human ECs for 8–16 h as an upstream priming agent, it remarkably stimulates the ability of ECs to respond to the downstream “Factor” combination of SCF, IL-3, SDF-1α, FGF-2 and insulin (Stratman et al., 2011; Bowers et al., 2020). VEGF priming followed by adding VEGF again, FGF-2 alone or their combination does not support EC tubulogenesis. Adding the “Factor” combination as an upstream priming stimulus followed by downstream addition of VEGF alone, FGF-2 alone or VEGF plus FGF-2 fails to support EC tube formation, so reversing the order of addition does not promote this process (Stratman et al., 2011; Bowers et al., 2020). Another important point is that VEGF priming of ECs stimulates the formation of EC tip cells, and accelerates the formation of lumens and tubes, but only following addition of the “Factors” (Bowers et al., 2020). Thus, activities attributed to VEGF *in vivo* such as driving tip cell sprouting behavior or inducing lumen expansion on its own are not supported by these new insights. Past interesting work demonstrated effects of VEGF on developmental vasculogenesis such as vessel fusion and lumen expansion in response to high levels of VEGF (Drake and Little, 1995, 1999). The new insights on VEGF priming suggest that such biological effects may also involve stimulation of downstream “Factor”-dependent signaling (Bowers et al., 2020). Also, key roles for EC integrin-ECM interactions as well as MT-MMP-dependent proteolysis as requirements for EC tip cell invasion during sprouting behavior are frequently overlooked, but they are crucial regulators of EC tip cell behavior. Finally, there is a marked acceleration of pericyte recruitment, proliferation, and capillary basement membrane deposition following VEGF priming of ECs and addition of the “Factors” during the capillary formative process (Bowers et al., 2020). Thus, VEGF priming accelerates all aspects of EC morphogenesis, while at the same time, stimulates pericyte responsiveness and recruitment to the more rapidly assembling EC tube networks leading to accentuated capillary basement membrane formation (Bowers et al., 2020). All of these events require the downstream presence of the “Factors.”

Why is VEGF able to accelerate vascular morphogenic and maturation events as an upstream primer in response to the downstream “Factors”? Recently, a detailed analysis of the VEGF priming pathway revealed key involvement of VEGFR2, RhoA, PKCα, and PKD2 induced signaling as critical to this priming process (Bowers et al., 2020). In addition, VEGF priming induced the EC expression of the “Factor” receptors, c-Kit, IL-3Rα, and CXCR4, and furthermore, induced the expression of EC-derived factors involved in pericyte recruitment including PDGF-BB, PDGF-DD, and HB-EGF (Bowers et al., 2020). Thus, VEGF priming appears to directly stimulate the ability of ECs to respond to the “Factors” through EC receptor upregulation to drive vascular morphogenesis, but also pericyte recruitment to the EC-lined tubes *via* greater production of the EC-derived factors that stimulate the pericyte recruitment process (Bowers et al., 2020). Overall, this work suggests that the biological influence of VEGF on vessel assembly, including basement membrane deposition, appears to be primarily mediated by its action as an upstream primer, and that other critical downstream “Factors” are the direct acting ones that stimulate vascular morphogenic and maturation events in conjunction with EC-ECM interactions.

### Growth factor and signaling control of mural cell recruitment during arteriogenesis

Based on the discussion above, an interesting question concerns the role of VEGF priming and the downstream “Factors” in the development of the arterial system. Of great importance is that VEGF was shown to be required for the development of arteries in the developing zebrafish as well as downstream Notch activation (Notch1 and Notch4 in ECs) (Lawson et al., 2001; Lawson et al., 2002). In developing embryos, the vascular remodeling to create the arterial system is dependent on flow forces (Lucitti et al., 2007; Udan et al., 2013), which also play a role in EC Notch activation, a stimulus that further suppresses EC proliferation (Fang et al., 2017). Data from work *in vitro* shows that inhibition of Notch activation using γ-secretase inhibitors leads to increase EC sprouting behavior (Sainson et al., 2005; Salvador and Davis, 2018). In our work, this increased EC sprouting response appears to be directly correlated with inhibition of EC lumen formation (Kim et al., 2017; Salvador and Davis, 2018), so Notch activation is important to form and stabilize EC lumens and tubes. In the above description of VEGF priming experiments, it is evident that there is a strong enhancement of pericyte recruitment following this priming stimulus, and thus, VEGF priming, and downstream “Factor” addition leads to rapid assembly of capillary-like structures. Could this assembly process be a developmental precursor to arteriogenesis, perhaps following application of flow forces? Flow forces should enhance Notch activation in ECs and might influence the conversion of pericytes into the vascular smooth muscle lineage. One study, by performing lineage tracing analysis, provided evidence that pericytes are the developmental precursors of vascular smooth muscle cells in the embryonic heart and kidney, a conversion process that required Notch3 (Volz et al., 2015). Interestingly, genetic deletion of Notch3 did not affect the pericyte lineage or their association with developing EC tube networks (Volz et al., 2015). It is important to determine how to create arterial vascular networks *in vitro* through the association of arterial EC tubes with vascular smooth muscle cells. Are differentiated vascular smooth muscle cells even capable of recruiting to EC tubes or are only pericytes capable of this recruitment? If the latter statement is true, pericytes once recruited to developing arteries would need to differentiate into vascular smooth muscle cells, while pericytes that have recruited to capillaries would remain pericytes. The molecular basis for how such a distinction in differentiation occurs represents an interesting question. An alternative possibility is that vascular smooth muscle cells arise from a different cell type that is capable of recruiting to EC-lined tubes during tissue vascularization. Thus, pericytes, which are functionally designed to recruit to EC tube networks, would be an ideal cell type to be the developmental precursor for VSMCs based on this ability. Future studies need to be performed to further uncover how VSMCs arise and how EC-VSMC interactions regulate the formation and organization of arteries and veins, where VSMCs represent the dominant mural cell type.

### Deficiencies in EC-pericyte interactions and capillaries play key pathogenic roles in major human diseases

It is becoming increasingly clear that capillary dysfunction and regression (i.e. rarefaction) are central pathogenic regulators of major human diseases. Such diseases include hypertension, ischemic tissue injuries including myocardial infarction and stroke, diabetes, heart failure, sepsis, neurodegenerative diseases, malignant cancers, vascular malformations, and aging. Diabetes is a disease whereby abnormalities in EC and pericyte interactions occur and this is a chronic disease state that can become progressively more severe with time. A key pathogenic feature of the disease is pericyte loss (Hammes, 2005), which has been attributed to activation of protein kinase C delta, and the induction of pericyte apoptosis (Geraldes et al., 2009). One of the more severe manifestations of the disease is diabetic retinopathy, with a spectrum of evident retinal injuries including those caused by excessive vascular permeability, and/or hemorrhage (Arboleda-Velasquez et al., 2015). In addition, capillary microaneurysms can manifest, which predispose to retinal hemorrhage (Arboleda-Velasquez et al., 2015). Diabetic capillaries also have abnormal basement membranes as observed by transmission electron microscopy, which are most likely a consequence of abnormal EC-pericyte interactions. Basement membrane degradation may be a pathogenic feature of this disease, which could liberate many growth factors and mediators as discussed earlier, that might allow for continued EC morphogenesis or accelerated regression of vessels. Overall, the capillary vasculature appears to be unstable with elements of formation and regression occurring simultaneously leading to clinically important consequences such as excessive retinal vessel permeability and hemorrhage, both which can contribute to vision loss, for example. The complexity of diabetes and its chronicity in humans, makes it difficult to accurately mimic the human disease using rodent models. This important disease needs to be modeled using the advanced 3D *in vitro* EC-pericyte model systems of today to add to our molecular understanding of why capillaries are abnormal in diabetes and perhaps develop new therapeutic approaches to treat the disease.

Two other key human disease states with abnormal pericyte associations with EC tubes are malignant cancers and a spectrum of different vascular malformations. In the case of malignant cancers, pericytes appear to become more loosely associated with capillaries, which appears to contribute to the unstable vasculatures within these tumors (Abramsson et al., 2002; Morikawa et al., 2002; Picoli et al., 2021). Like with diabetes, increased vascular permeability and hemorrhage are observed along with focal tumor necrosis, all of which suggest that the capillaries are dysfunctional. Since many malignant tumors produce high levels of VEGF (Dvorak et al., 2011), it has been suggested that VEGF can affect pericyte association with capillaries. Blockade of VEGF or its receptor in these tumor microenvironments does appear to enhance pericyte association with the capillaries and improve perfusion into the tumor, a process that has been termed tumor vessel normalization (Jain, 2005; Goel et al., 2012). These are very interesting observations, and much more work is needed to understand exactly how these abnormalities arise and how they could be potentially therapeutically corrected. As mentioned previously, transient VEGF treatment (i.e. a priming stimulus for 16 h) of ECs leads to upregulation of PDGF-BB, and PDGF-DD, and HB-EGF, which promotes pericyte association with EC tubes, increased pericyte proliferation, and enhanced basement membrane deposition, all steps that drive capillary assembly and eventual stabilization (Bowers et al., 2020). Once capillaries have assembled and stabilized, the activities of PDGF-BB, PDGF-DD, ET-1, HB-EGF and TGFβ1 (which together drive pericyte recruitment, proliferation and basement membrane assembly) may need to be suppressed, so that continued vessel formation does not occur. In contrast, chronic VEGF exposure might enhance the expression and continued release of these EC-derived pericyte activating factors, which induce pericytes to leave capillaries, and invade in between capillaries, creating vessel instability. Such events might also account for pericyte loss in diabetes, where pericytes could be induced to migrate off the capillary abluminal surface. Also, chronic exposure to VEGF (and probable exposure to the EC “Factors”) might further promote EC tube morphogenesis, such as lumen expansion, and basement membrane degradation to further destabilize the capillary walls. More work is needed to elucidate the biology of why capillaries are destabilized in the malignant tumor microenvironment.

Abnormalities in EC-pericyte assembly are also apparent in various vascular malformation syndromes where fewer pericytes/mural cells are observed to be associated with the abnormal vasculature (Cai et al., 2019). In particular, they have been observed in arteriovenous (AVM), venous (VM) and cerebral cavernous malformations (CCM). Lower levels of PDGF-BB have been implicated as a cause of these deficiencies (Redondo et al., 2010). These vascular malformations have in common the presence of activating mutations in key signal transduction molecules including k-Ras with AVM, Tie2 with VM, and PIK3CA with CCM(Limaye et al., 2009; Al-Olabi et al., 2018; Nikolaev et al., 2018; Cai et al., 2019; Ren et al., 2021). CCM also have familial or sporadic mutations in either the CCM1, CCM2 or CCM3 genes (Lampugnani et al., 2017; Wetzel-Strong et al., 2017). Genetic knockouts of each of these CCM genes during vascular development led to EC lumen formation defects (Kleaveland et al., 2009; Whitehead et al., 2009; Lampugnani et al., 2017). VEGF and VEGFR2-dependent signaling have been shown to play a role in previous work on the pathogenesis of a variety of vascular anomalies (Park et al., 2021; Queisser et al., 2021; Snodgrass et al., 2021). An interesting question concerns the potential role of VEGF priming and downstream “Factor”-dependent signaling in the morphogenic manifestations and abnormalities observed in the various vascular anomalies.

In new work from our laboratory, ECs expressing activated k-RasV12 (mimicking ECs within AVM lesions), show markedly accelerated lumen formation mechanisms and form excessively widened tube networks in 3D matrices (Sun et al., 2022). However, these abnormal EC tube networks exhibit strongly reduced pericyte recruitment and basement membrane deposition consistent with findings *in vivo* in a variety of vascular malformations (Sun et al., 2022). Because multiple EC-derived factors participate in pericyte recruitment to EC-lined tubes during capillary assembly (Kemp et al., 2020), more work needs to be performed to determine the detailed mechanistic reasons why pericytes don’t recruit well to malformed vessels expressing unique sets of genetic mutations. It is probable that the answers here will be complex since EC morphogenesis is abnormal (and unique to the particular malformation syndrome), in combination with deficiencies in the synthesis, secretion (i.e. availability) or stability of the key EC-derived factors necessary for pericyte recruitment, proliferation and pericyte-induced basement membrane deposition (Figure 3). Each case will need to be examined separately in detail because the unique sets of activating mutations or other mutations found in ECs, which underlie these syndromes, will probably regulate these pericyte factors in distinct ways. This information will be necessary to develop novel and effective therapeutic options to treat both the EC morphogenic abnormality as well as the reasons for the EC-mural cell interaction defect.

## Conclusions

In this review, we have discussed the fundamental role of pericytes in the assembly, maturation, and stabilization of capillary tube networks. Work over the past several decades using both *in vivo* and *in vitro* experimental approaches have led to many key answers regarding the signaling basis for how ECs form tube networks, and how specific sets of growth factors and peptides separately control ECs and pericytes to co-assemble in a polarized manner in 3D extracellular matrix environments. There is remarkable coordination of EC tube network assembly with the recruitment of pericytes along the EC abluminal surface, and then deposition of the capillary basement membrane matrix, a necessary step in the maturation and stabilization of capillaries. Abnormalities and loss of capillaries is a predisposing factor in the development of major human diseases including diabetes, malignant cancers, vascular malformations, and the progression of aging. Despite their critical importance for tissue development and health, not enough emphasis has been placed on comprehensively understanding normal capillary biology as well as capillary dysfunction and regression. For example, capillary regression (i.e. rarefaction) is a fundamental regulator of major human disease states, and only recently, have important advances been made in elucidating key pro-regressive molecules, signal transduction pathways controlling EC tube regression, and pharmacologic approaches to block these processes (Koller et al., 2020). Interestingly, in these studies, the major pro-inflammatory mediators, IL-1β, TNFα, and thrombin, were found to promote EC tube disassembly and regression and EC apoptosis, individually and in combination (Koller et al., 2020). A fascinating finding from this work is that despite the marked loss of EC tubes and individual ECs, these same pro-regressive molecules, did not cause pericyte loss, and instead led to apparent pericyte proliferation and activation (Koller et al., 2020). It is very important to investigate this latter phenomenon in detail to evaluate such pericyte responses resulting from capillary dysfunction and regression which may directly contribute to the underlying pathogenesis of key human diseases.
